# TLR2-independent induction and regulation of chronic intestinal inflammation

**DOI:** 10.1002/eji.200939669

**Published:** 2009-11-30

**Authors:** Olivier Boulard, Mark J Asquith, Fiona Powrie, Kevin J Maloy

**Affiliations:** Sir William Dunn School of Pathology, University of OxfordOxford, UK

**Keywords:** Inflammatory bowel disease, Intestinal immunity, Mucosal immunity, TLR, Treg

## Abstract

Interactions between the intestinal microflora and host innate immune receptors play a critical role in intestinal homeostasis. Several studies have shown that TLR2 can modulate inflammatory responses in the gut. TLR2 signals enhance tight junction formation and fortify the epithelial barrier, and may play a crucial role in driving acute inflammatory responses towards intestinal bacterial pathogens. In addition, TLR2 agonists can have direct effects on both Th1 cells and Treg. To define the role of TLR2 in the induction and regulation of chronic intestinal inflammation we examined the effects of TLR2 deletion on several complementary models of inflammatory bowel disease. Our results show that TLR2 signals are not required for the induction of chronic intestinal inflammation by either innate or adaptive immune responses. We further show that TLR2^−/−^ mice harbor normal numbers of Foxp3^+^ Treg that are able to suppress intestinal inflammation as effectively as their WT counterparts. We also did not find any intrinsic role for TLR2 for pathogenic effector T-cell responses in the gut. Thus, in contrast to their role in acute intestinal inflammation and repair, TLR2 signals may have a limited impact on the induction and regulation of chronic intestinal inflammation.

**See accompanying commentary:** http:/dx.doi.org/10.1002/eji.200940232

## Introduction

Dysregulation of immune responses toward the intestinal microflora is thought to underlie the chronic pathology observed in inflammatory bowel diseases (IBD), such as Crohn's disease (CD) and ulcerative colitis 1, 2. Accumulating evidence indicates that intestinal homeostasis is influenced by a variety of factors, particularly those involved in host–microflora interactions 1, 2. PRR, such as TLR and NOD-like receptors (NLR), constitute the major innate receptors used to recognize foreign organisms and to initiate innate and adaptive responses 3, 4. A role for PRR in IBD was highlighted by the discovery that polymorphisms of the nucleotide-binding oligomerization domain containing protein 2 (*NOD2*) (*CARD15*) gene were associated with increased susceptibility to CD 5, 6. *NOD2* encodes a cytoplasmic NLR involved in the recognition of bacterial peptidoglycan; however, the precise mechanisms through which *NOD2* polymorphisms predispose to CD have remained elusive 7, 8. Although additional studies have suggested that TLR gene polymorphisms may also influence susceptibility to IBD 9–11, investigating the functional roles of PRR is challenging, partly because PRR may be expressed by a variety of cell types within the gastrointestinal tract 12.

Recent studies have revealed that TLR signals can influence intestinal homeostasis in several ways 12. TLR signals play a critical role in repair and restitution of the intestinal epithelium following tissue damage. For example, mice lacking TLR2 or TLR4 or the MyD88 adaptor protein involved in TLR signal transduction, exhibited increased susceptibility to the chemically induced dextran sulfate sodium (DSS) model of colitis 13–15. Furthermore, administration of various TLR agonists has been shown to prevent or ameliorate DSS colitis 12. TLR signaling leads to activation of NF-κB and a basal level of NF-κB expression in intestinal epithelial cells is critical for maintaining epithelial integrity and preventing chronic inflammation 16, 17. Conversely, TLR signals can also drive pathogenic immune responses in the gut. For example, the spontaneous T-cell-mediated colitis observed in IL-10^−/−^ mice did not arise in MyD88^−/−^IL-10^−/−^ mice 18. Similarly, administration of TLR agonists may exacerbate ongoing colitis 19, 20, while antagonists may attenuate intestinal inflammation 21.

T cells can also express TLR 22 and diverse TLR agonists have been reported to directly enhance T-cell responses 23–25. In addition, naturally occurring CD4^+^CD25^+^ Treg may also be modulated by TLR signals 26. Treg play a critical role in inhibiting intestinal inflammation and a variety of direct effects of TLR agonists on Treg have been described, including enhancement or abrogation of their suppressive activities 27–31.

Several findings suggest that TLR2 may be a particularly important contributor to intestinal homeostasis. Administration of TLR2 agonists ameliorated DSS colitis by activating cryoprotective responses in intestinal epithelial cells and enhancing tight junction formation 14, 15, 32. Furthermore, TLR2 agonists stimulated Treg proliferation that was accompanied by a transient loss of suppressive function *in vitro* 28, 30. However, other TLR2 agonists have been reported to enhance Treg suppression 31 and TLR2^−/−^ mice were reported to harbor reduced numbers of Treg 33. Conversely, TLR2 agonists can also provide co-stimulatory signals for conventional T cells 25 and may directly trigger Th1 effector responses 34. Similarly, TLR2 was required for disease in a model of T-cell transfer colitis that suggested that a crucial function of NOD2 is to inhibit TLR2-driven pathogenic Th1 responses in the gut 35, 36. Therefore, under different experimental conditions, TLR2 signals may exacerbate or attenuate intestinal inflammation.

Here we have comprehensively assessed the role of TLR2 signals in different cellular compartments during the induction and regulation of chronic intestinal inflammation. To assess the role of TLR2 signals in pathogenic effector T cells and in Treg we used the T-cell transfer model of IBD 37, whereas IBD models based on infection with the intestinal bacterium *Helicobacter hepaticus* 38–40 allowed us to examine the role of TLR2 in innate immune-mediated IBD. *In vitro* studies implicated TLR2 in the induction of pro-inflammatory responses by *H. hepaticus* and also by the closely related human pathogen *Helicobacter pylori* 41, 42. However, our results demonstrate that TLR2 is not required for the induction of chronic intestinal inflammation by either innate or adaptive immune responses. We further show that TLR2 is dispensable for the development and suppressive function of CD4^+^CD25^+^Foxp3^+^ Treg *in vivo*. We also did not find any intrinsic role for TLR2 for the development of pathogenic effector T-cell responses in the gut. These results suggest that intestinal bacteria trigger chronic inflammation through the stimulation of other PRR pathways and that modulation of TLR2 signals may have limited impact during the chronic phase of intestinal inflammation.

## Results

### TLR2 expression on host APC is not required for the induction or regulation of T-cell-dependent colitis

**Figure 1:**
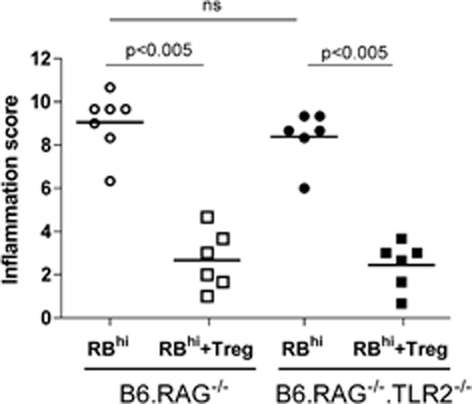
T–cell-mediated colitis and its regulation are independent of TLR2 expression by host APC. Cohorts of B6.RAG^−/−^ and B6.RAG^−/−^TLR2^−/−^ mice were reconstituted with 4×10^5^ WT CD4^+^CD45RB^high^ T cells alone or with 2×10^5^ CD4^+^CD25^+^ Treg by i.p. injection. After 8–10 wk colonic pathology was assessed histologically; *n*=6–7 mice *per* group. Horizontal lines represent group means and differences were assessed using the Mann–Whitney *U*-test; ns, not significant.

To examine whether TLR2 expression by host APC was essential for the induction of pathogenic T-cell responses directed against the intestinal microflora, we utilized a well-characterized T-cell transfer model of colitis 37. As shown in Fig. 1, adoptively transferred C57BL/6 (B6) WT naYve CD4^+^CD45RB^hi^ T cells elicited severe colitis in B6.RAG^−/−^ or B6.RAG^−/−^.TLR2^−/−^ recipients, with no differences in either severity or incidence of disease. This model also allowed us to address whether TLR2 expression on host APC played a role in the regulation of colitis by naturally occurring CD4^+^CD25^+^Foxp3^+^ Treg 37, 43. However, we found that B6 WT CD4^+^CD25^+^Foxp3^+^ Treg efficiently suppressed T-cell-mediated colitis in both B6.RAG^−/−^ or B6.RAG^−/−^.TLR2^−/−^ recipients (Fig. 1). Thus, TLR2 expression on host APC is not required for either effector T-cell or Treg activity in the intestine.

### Intrinsic TLR2 signals are not required for effector T-cell or Treg activity *in vivo*

**Figure 2:**
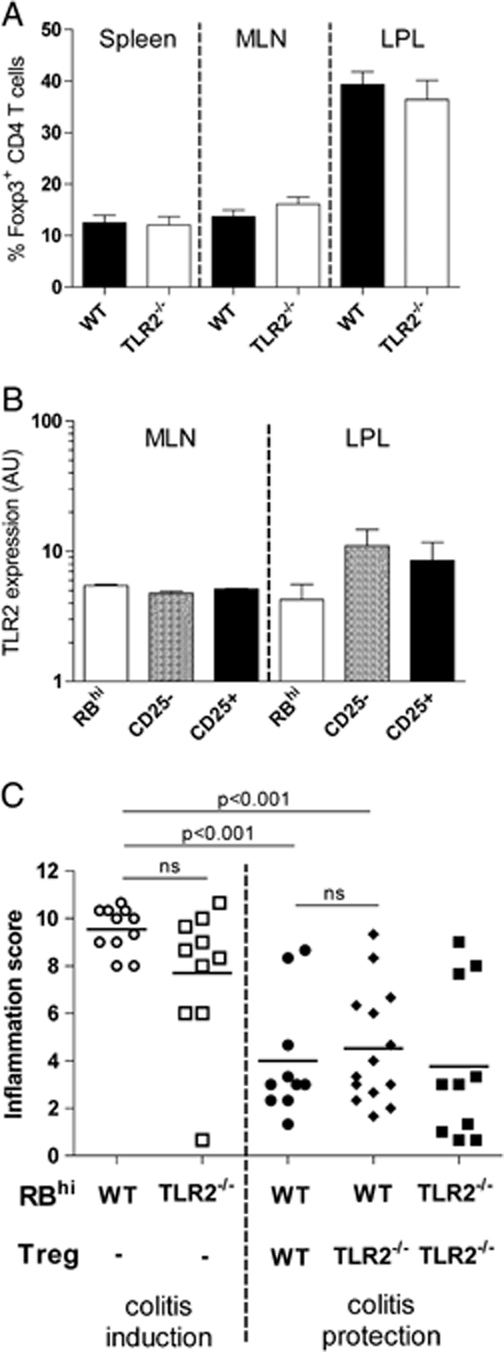
T-cell intrinsic TLR2 signals are not required for effector T-cell or Treg functions *in vivo*. (A) CD4^+^ T cells from the spleen, MLN and colonic LPL were analyzed for Foxp3 expression by flow cytometry. Data represents group means (±SEM) from two pooled independent experiments (*n*=8–10 mice *per* group). (B) NaYve CD45RB^hi^ (RB^hi^), memory CD25^−^CD45RB^lo^ (CD25^−^) and Treg CD25^+^CD45RB^lo^ (CD25^+^) CD4^+^ T cells were sorted from the MLN and LPL of B6 WT mice (*n*=5) and analyzed for TLR2 gene expression by Q-PCR. Data represent means±SEM normalized relative to HPRT. (C) B6.RAG^−/−^ mice were reconstituted with either 4×10^5^ WT or TLR2^−/−^ CD4^+^CD45RB^hi^ T cells alone (colitis “induction”) or concurrently with 2×10^5^ WT or TLR2^−/−^ CD4^+^CD25^+^ Treg (colitis “protection”). After 8–10 wk colonic pathology was assessed histologically. Each symbol represents a single animal and the data represents pooled results of two independent experiments (*n*=10–14 mice *per* group). Horizontal lines represent group means and differences were assessed using the Mann–Whitney *U*-test; ns, not significant.

Recent studies have reported that T cells may express TLR2 and that TLR2 signals can modulate the activities of both conventional T cells 25, 34 and Treg 28, 30, 31. As a marked decrease in peripheral CD4^+^CD25^+^ T cells was reported in TLR2^−/−^ mice 33, we first assessed the proportions of Treg in lymphoid organs and in the intestinal mucosa of B6.TLR2^−/−^ mice using intracellular staining for the Foxp3 transcription factor 44. We observed equivalent percentages of CD4^+^Foxp3^+^ T cells in the spleen and MLN from B6 WT and B6.TLR2^−/−^ mice (Fig. 2A). In addition, although we observed higher frequencies of CD4^+^Foxp3^+^ T cells amongst colonic lamina propria leukocytes (LPL), these increases were equivalent in both B6 WT and B6.TLR2^−/−^ mice (Fig. 2A). Thus, TLR2 signals are not required for the development or peripheral maintenance of CD4^+^Foxp3^+^ Treg.

We next used quantitative PCR (Q-PCR) to assess the levels of TLR2 expression on different subpopulations of CD4^+^ T cells. Sorting based on expression of CD45RB and CD25 facilitates sub-fractionation of CD4^+^ T cells into naYve (CD45RB^hi^), memory (CD45RB^lo^CD25^−^) and regulatory (CD45RB^lo^CD25^+^) populations 37. As illustrated in Fig. 2B, we found equivalent levels of TLR2 mRNA expression in all CD4^+^ T-cell populations isolated from the MLN. In addition, although there was a modest increase in TLR2 expression by memory (CD45RB^lo^CD25^–^) and regulatory (CD45RB^lo^CD25^+^) CD4^+^ T cells isolated from the colonic lamina propria, these differences were not statistically significant (Fig. 2B). Thus, we could not detect enhanced TLR2 expression by CD4^+^ Treg populations in the MLN or LPL.

We next examined whether T-cell intrinsic TLR2 signals were required for the functional activities of effector T cells or Treg in the intestine. Adoptive transfer of naYve CD4^+^CD45RB^hi^ T cells isolated from either B6 WT or B6.TLR2^–/–^ mice elicited severe colitis in B6.RAG^–/–^ recipients, with no significant differences in either severity or incidence of disease (Fig. 2C). Furthermore, co-transfer of CD4^+^CD25^+^ Treg isolated from B6.TLR2^–/–^ mice inhibited colitis development to a similar degree as B6 WT CD4^+^CD25^+^ Treg (Fig. 2C). Similar suppression of colitis was observed when both naYve CD4^+^CD45RB^hi^ T cells and CD4^+^CD25^+^ Treg isolated from B6.TLR2^–/–^ mice were co-transferred into B6.RAG^–/–^ recipients (Fig. 2C).

In order to look for more subtle effects of TLR2 on intestinal T-cell responses, we monitored the disease kinetics, using weight loss as an indicator of disease onset 37. We found that B6.RAG^–/–^ recipients of naYve CD4^+^CD45RB^hi^ T cells isolated from either B6 WT or B6.TLR2^–/–^ mice began to lose weight around 5–6 wk post T-cell transfer, at the same time as clinical symptoms such as diarrhea became apparent (Supporting Information Fig. 1). Similarly, CD4^+^CD25^+^ Treg isolated from B6.TLR2^−/−^ mice seemed to function as efficiently as those from B6 WT mice, as they promoted equivalent levels of weight gain in B6.RAG^−/−^ recipients that had been co-transferred with naYve CD4^+^CD45RB^hi^ T cells (Supporting Information Fig. 1). These results indicate that T-cell intrinsic TLR2 signals are not required for the development or functional activities of pathogenic effector T cells or Treg *in vivo*.

### *H. hepaticus* triggered innate immune IBD is TLR2-independent

**Figure 3:**
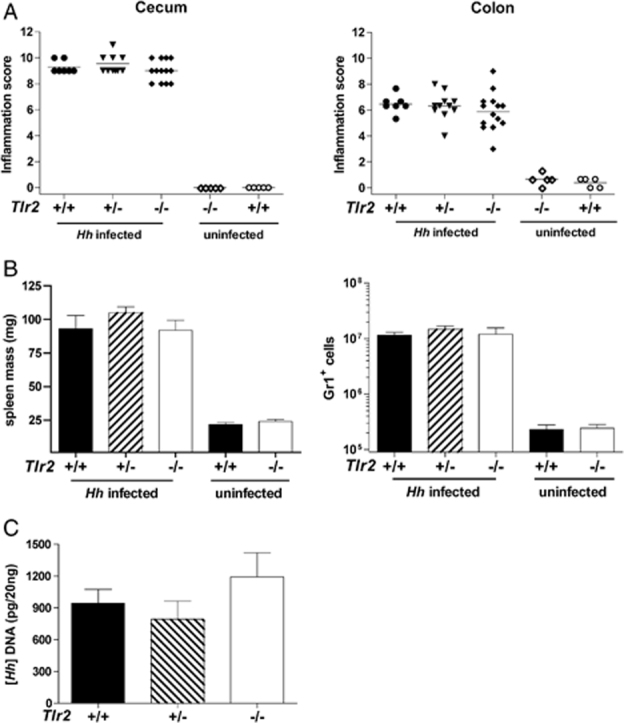
*H. hepaticus*-induced innate immune IBD is TLR2-independent. (A) Cohorts of 129SvEv.RAG^−/−^TLR2^+/+^, 129SvEvRAG^−/−^TLR2^+/−^ and 129SvEv.RAG^−/−^TLR2^−/−^ mice were infected with *H. hepaticus* and cecal and colonic pathology assessed 6–8 wk later. Each symbol represents a single animal and the data represent pooled results from three independent experiments (*n*=5 uninfected controls and *n*=7–14 mice for experimental groups). Horizontal lines represent group means. (B) Spleen mass was measured and granulocytes (Gr1^Hi^CD11b^Int^CD11c^−^ cells) enumerated by FACS. Graphs represent group means±SEM. (C) Cecal contents were collected and *H. hepaticus* DNA quantified using Q-PCR. Graphs represent group means±SEM.

To address whether TLR2 signals were essential for activation of innate inflammatory pathways in the intestine, we utilized a model where chronic intestinal inflammation is mediated by innate immune activation in 129.RAG^−/−^ mice, following infection with the intestinal bacterium *H. hepaticus* 39. In addition, previous studies have suggested that TLR2 plays a key role in the induction of inflammatory responses to chronic *Helicobacter* infections 41, 42. We therefore generated 129.RAG^−/−^.TLR2^−/−^ mice and infected these, alongside control littermates, with *H. hepaticus*. As shown in Fig. 3A we found that TLR2 genotype did not influence *H. hepaticus*-induced innate immune typhlocolitis, with infected 129.RAG^−/−^.TLR2^−/−^ mice exhibiting a similar degree of inflammation in the cecum and colon as infected 129.RAG^−/−^.TLR2^+/+^ littermates. *H. hepaticus*-induced innate immune typhlocolitis is accompanied by systemic activation of the innate immune system 39 and this was also unaffected by TLR2 genotype, as infected 129.RAG^−/−^.TLR2^−/−^ mice had similar degrees of splenomegaly and granulocyte accumulation as infected 129.RAG^−/−^.TLR2^+/+^ littermates (Fig. 3B). Finally, using a Q-PCR assay 39, 45, we found equivalent *H. hepaticus* colonization levels in 129.RAG^−/−^.TLR2^−/−^ mice and 129.RAG^−/−^.TLR2^−/−^ mice (Fig. 3C). Together, these results indicate that TLR2 signals are not required for activation of innate immune responses by *H. hepaticus* and that TLR2 deficiency does not result in increased colonization by *H. hepaticus*.

### TLR2 is dispensable for the induction of IBD in immune-competent mice

**Figure 4:**
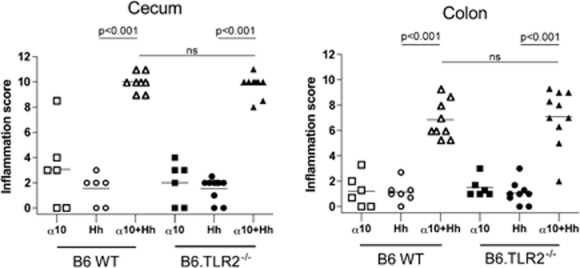
Chronic intestinal inflammation in normal mice is TLR2-independent. B6 WT and B6.TLR2^−/−^ mice were infected orally with *H. hepaticus* and treated weekly with 1 mg anti-IL10R mAb i.p. (α10+Hh). On day 28, cecal and colonic pathology were assessed histologically. Control groups received *H. hepaticus* (Hh) or anti-IL10R alone (α10). Each symbol represents a single animal and data represent combined results from two independent experiments (*n*=6–10 mice for controls; *n*=9–10 for Hh+antiIL10R-treated groups). Horizontal bars represent group means and differences were assessed using the Mann–Whitney *U*-test; ns, not significant.

To investigate the role of TLR2 in intestinal pathology in non-lymphopenic mice we used a recently developed IBD model, where disease is induced in WT B6 mice by infection with *H. hepaticus* together with concomitant administration of anti-IL-10R antibody 40. Although single treatments (*H. hepaticus* infection or anti IL-10R alone) elicited minimal inflammatory responses, the combination of *H. hepaticus* infection with anti-IL-10R antibody administration elicited severe typhlocolitis, with equivalent levels of intestinal inflammation observed in both B6 WT and B6.TLR2^−/−^ mice (Fig. 4). Analyses of colonization showed that there were similar levels of *H. hepaticus* present in all infected groups (data not shown). Thus, TLR2 is not required for the induction of IBD pathology in normal immune competent mice and does not appear to have a significant influence on colonization of the large intestine by *H. hepaticus*.

## Discussion

Although TLR2 signals have been implicated in both deleterious and host protective immune responses to pathogens, their significance in intestinal regulation and inflammation remains poorly defined. To this end, we exploited various TLR2 knock-out mice to define the role of TLR2 signals in chronic innate and adaptive inflammatory responses in the intestine.

Using an innate immune-mediated IBD model triggered by infection with *H. hepaticus* we found that 129RAG^−/−^.TLR2^−/−^ mice developed bacterially driven typhlocolitis comparable to that of TLR2-competent controls. This was somewhat unexpected because *H. hepaticus* has been reported to activate macrophages and dendritic cells in a TLR2-dependent manner *in vitro* 41 and TLR2 signals have also been reported to contribute to inflammatory cytokine production in response to *H. pylori* 42. In addition, up-regulated expression of TLR2 has been described in inflamed human colonic tissue 46 and dysregulated TLR2-mediated inflammatory responses have been proposed to underlie the strong association between *NOD2* polymorphisms and CD 8. Thus, TLR2-deficiency prevented the induction of T-cell-mediated colitis in NOD2^−/−^ mice 36 and a recent study reported that NOD2 may down-regulate multiple TLR responses 47. Although these studies implicate a colitogenic role for TLR2 signals in the absence of NOD2, our findings indicate that TLR2 signals are not absolutely required for the activation of chronic inflammatory responses towards intestinal bacteria.

The reported requirement for MyD88 signals in microflora-induced IBD 18, 48, suggests that alternative TLR signals may drive *H. hepaticus*-induced disease. However, other reports have provided evidence that *H. hepaticus* has evolved means to minimize the activation of TLR pathways. For example, *H. hepaticus* LPS has been reported to actively suppress TLR4-mediated innate responses 49 and *Helicobacter* flagellins do not activate TLR5 50. Although the role of TLR9 in *H. hepaticus*-mediated intestinal inflammation remains undefined, several studies reported that TLR9-mediated recognition of bacterial DNA motifs exerted anti-inflammatory effects in the intestine 51–53, while others suggested that TLR9 signals helped to perpetuate chronic intestinal inflammation 19, 54.

Alternatively, it is possible that additional families of PRR, such as the NLR family 4, trigger innate inflammatory responses against gastro-intestinal *Helicobacter spp*. This possibility is supported by observations that NOD1^−/−^ mice exhibited enhanced susceptibility to *H. pylori* infection 55 and by our previous findings that *H. hepaticus*-driven typhlocolitis was associated with a marked increase in colonic IL-1β levels 38. In addition, genetic polymorphisms influencing IL-1β production have been implicated in susceptibility to gastric cancer in *H. pylori*-infected individuals 56.

Consistent with its non-essential role in innate immune typhlocolitis, we found that TLR2 signals in host APC were not required to induce pathogenic T-cell responses following transfer of naïve T cells into B6.RAG^−/−^.TLR2^−/−^ mice or following anti-IL-10R antibody treatment of *H. hepaticus*-infected B6.TLR2^−/−^ mice. As pathology is driven by intestinal bacteria 40, 57, we conclude that TLR2-mediated stimulation of APC by microflora constituents is mechanistically redundant for the induction of colitogenic T-cell responses. There is currently a great deal of interest on the potential functional roles of TLR expressed by T cells. TLR2 is expressed on activated T cells 22 and TLR2 agonists have been implicated in the direct co-stimulation of CD4^+^ T cells *in vitro* 25, 34. However, we found T-cell-intrinsic TLR2 signals to be dispensable for the activation and recruitment of effector CD4^+^ T cells *in vivo*, as evidenced by the potent colitogenic potential of TLR2^−/−^ naYve T cells and by the severe typhlocolitis induced by anti-IL-10R antibody treatment of *H. hepaticus*-infected B6.TLR2^−/−^ mice. Using the T-cell transfer IBD model, it was recently reported that MyD88-deficient T cells had proliferative defects, were refractory to *ex vivo* restimulation and exhibited an attenuated ability to induce colitis 58, 59. We propose that this effect is independent of TLR2 and may reflect activation of alternative MyD88-dependent signaling receptors on T cells, such as additional TLR, IL-1/IL-18R and IFN-γR.

Although recent studies implicated TLR2 in Treg function, we found no influence of TLR2 on the frequency of Foxp3^+^ Treg in the spleen, MLN or LPL. This contrasts with earlier reports of a significant decrease of CD4^+^CD25^+^ Treg in TLR2^−/−^ mice 33, although Foxp3 expression was not evaluated. This differential may be explained by the choice of population marker or differences in microbial exposure of respective experimental mice, which may have altered the frequency of activated CD25^+^ effector T cells. Importantly, we found no functional requirement for TLR2 in Treg function, as TLR2^−/−^ Treg suppressed colitis as effectively as WT Treg. Our results contrast somewhat with previous studies that reported *in vitro* stimulation with selective TLR2 agonists induced expansion of Treg and a transient loss of suppressive function 28, 30. Therefore, although TLR2 signals may augment Treg-proliferative responses and contribute to overcoming constitutive Treg activity under some circumstances, T-cell intrinsic TLR2 signals are clearly not required for the expression of functional Treg activity *in vivo*.

Our results also argue against an essential protective role for TLR2 signaling in intestinal inflammation, despite evidence that TLR2 signals may regulate epithelial cell proliferation and tight junction formation following acute inflammatory stress, elicited by DSS administration or *Citrobacter rodentium* infection 15, 60. Our study adopted models of chronic intestinal pathology, marked by non-resolving inflammation lasting weeks rather than days, and TLR2-deficiency had no impact. This suggests that TLR2-mediated promotion of epithelial repair is largely context dependent, operating at a level of inflammatory stress greater than that induced in our colitis models. Finally, our observations that *H. hepaticus* colonization levels were equivalent in WT and TLR2^−/−^ mice, together with similar findings in mice infected with *C. rodentium* 60, suggest that TLR2 signals do not exert a protective effect in the intestinal epithelium by limiting the extent of bacterial growth.

In conclusion, despite evidence that TLR2 signals can modulate intestinal homeostasis through direct effects on epithelial cell and indirect effects on Treg, our results indicate that TLR2 signals are entirely dispensable for the induction and regulation of chronic intestinal pathology. Therefore modulation of TLR2 activation may be of limited therapeutic benefit during chronic intestinal inflammation and future studies should focus on alternative PRR pathways that may play a non-redundant role in IBD pathogenesis.

## Materials and methods

### Mice

TLR2^−/−^ mice on B6 background 61 were crossed with B6.RAG1^−/−^ mice to generate B6.RAG^−/−^.TLR2^−/−^ mice. TLR2^−/−^ mice were derived onto the 129S6 background by iterative backcrossing of B6.TLR2^−/−^ with 129S6.RAG2^−/−^ mice utilizing a marker-assisted, “speed congenic” breeding method 62, 63. A total of 63 polymorphic micro-satellite markers, evenly spaced on each chromosome, were used to screen the offspring at each generation. The final 129.TLR2^−/−^.RAG2^−/−^ and 129.TLR2^−/−^ lines were established after the 7th backcross and TLR2^+/−^ and TLR2^+/+^ littermate controls were used in all experiments. Mice were maintained in accredited specific pathogen-free facilities and experiments conducted in accordance with the UK Scientific Procedures Act (1986).

### Bacteria

*H. hepaticus* NCI-Frederick isolate 1A (strain 51449) was grown on blood agar plates containing trimethoprim, vancomycin and polymixin B (Oxoid) under microerophilic conditions as described previously 39. Mice were fed three times on alternate days with *H. hepaticus* (∼10^8^ CFU) by oral gavage.

### Induction of IBD in immune-competent mice

IBD was induced in immune-competent B6 WT or B6.TLR2^−/−^ mice by infection with *H. hepaticus* and concomitant treatment with 1 mg of monoclonal anti-IL10R antibody i.p. on days 0, 7, 14 and 21 and sacrificed 1 wk later 40.

### Induction and prevention of T-cell transfer colitis

T-cell subsets were isolated from spleens of B6 or B6.TLR2^−/−^ mice, as described previously 37, 43. In brief, CD4^+^ T cells were stained with FITC anti-CD45RB, PE anti-CD25 and PerCP-Cy5.5 anti-CD4 (BD Biosciences) and naYve (CD4^+^CD25^−^CD45RB^hi^), memory (CD4^+^CD25^−^CD25RB^lo^) and regulatory (CD4^+^CD25^+^CD25RB^lo^) T-cell populations were purified (∼99%) by cell sorting (MoFlo; Dakocytomation). For colitis induction, age- and sex-matched cohorts of B6.RAG1^−/−^ or B6.RAG1^−/−^.TLR2^−/−^ mice received 4×10^5^ naYve CD4^+^CD45RB^hi^ T cells i.p.; for colitis prevention mice were additionally injected with 2×10^5^ CD4^+^CD25^+^CD45RB^lo^ T cells i.p. Disease onset was monitored by weighing the mice and by monitoring clinical symptoms, such as diarrhea and piloerection. Mice were sacrificed 8 wk post transfer or when they had lost ≥20% of their initial weight.

### Assessment of intestinal inflammation

Mice were sacrificed at the indicated time points and samples from cecum and proximal, mid, and distal colon were immediately fixed in buffered 10% formalin. 4–5 μm paraffin-embedded sections were stained with hematoxin and eosin, and inflammation was assessed using a modified version of a previously described scoring system 39, 43. In brief, each sample was graded semi-quantitatively (from 0–3) for the four following criteria: degree of epithelial hyperplasia and goblet cell depletion; leukocyte infiltration in the lamina propria; area of tissue affected; markers of severe inflammation (submucosal inflammation and/or crypt abscesses). Scores were added to give an overall inflammation score for each section (0–12). Ceca and colons were assessed separately, in a blinded fashion. The colonic score represents the average of individual scores from the sections of proximal, mid and distal colon.

### Flow cytometry

Aliquots of ∼1×10^6^ spleen cells in HBSS/0.1% BSA (Sigma Aldrich) were incubated for 30 min with FcR block (eBioscience) at 4°C, and surface staining steps performed for 20 min at 4°C with a panel of monoclonal antibodies as follows: biotinylated anti-DX5, biotinylated anti-Gr1, PE anti-CD11c and APC anti-CD11b (all obtained from BD Bioscience). Biotinylated antibodies were detected with streptavidin-conjugated PerCP or APC (BD Bioscience). Cells were fixed in 2% paraformaldehyde before acquisition. For intracellular Foxp3 staining, samples were fixed with eBioscience Fix/Perm Buffer following surface staining with APC anti-CD4 as above. Cells were subsequently stained with FITC anti-Foxp3 according to the manufacturer's instructions. Cells were acquired using a FACSCalibur flow cytometer (Becton Dickinson) and analyzed with FlowJo software (Treestar).

### Quantitation of TLR2 expression using Q-PCR

Spleen cells, MLN cells and LPL were isolated from B6 WT mice as previously described 39, 43. NaYve (CD4^+^CD25^−^CD45RB^hi^), memory (CD4^+^CD25^−^CD25RB^lo^) and regulatory (CD4^+^CD25^+^CD25RB^lo^) T-cell subpopulations were purified (∼99%) by cell sorting as described above. RNA was purified using an RNAeasy kit (QIAGEN) and cDNA synthesis was performed using a reverse transcriptase kit (Superscript III) with Oligo dT (both from Invitrogen). Q-PCR were performed using Quantitect Primer Assays with SYBR green PCR mastermix (both from QIAGEN). cDNA samples were assayed in triplicate using a Chromo4 detection system (MJ Research) and TLR2 gene expression levels for each individual sample were normalized to hypoxanthine phosphoribosyl-transferase (HPRT). Mean relative gene expression was calculated using the 2^−ΔC(T)^ method, as previously described 38–40.

### Quantitation of *H. hepaticus* colonization

Mouse cecal contents were collected upon sacrifice and DNA extracted using a DNA Stool Kit (QIAGEN). Q-PCR was used with primers specific for *H. hepaticus* cdtB gene 39, 45, using a Chromo4 detection system.

### Statistical analysis

For comparison of histology scores, Q-PCR data, spleen mass and cell counts, the non-parametric Mann–Whitney test was used (Prism 5, Graphpad Software). *p*-Values below 0.05 were deemed statistically significant.

## Supplementary material

Detailed facts of importance to specialist readers are published as ”Supporting Information”. Such documents are peer-reviewed, but not copy-edited or typeset. They are made available as submitted by the authors.
