# Supplementary material for: TLR2-independent induction and regulation of chronic intestinal inflammation
Source: Eur J Immunol. 2009 Nov 30;40(2):516–24. doi: 10.1002/eji.200939669 (PMC3812679; doi:10.1002/eji.200939669)
Supplement: Supplementary file 1 [file eji0040-0516-SD1.pdf]

European Journal of Immunology

Supporting Information

for

DOI 10.1002/eji.200939669

TLR2-independent induction and regulation of chronic intestinal inflammation

Olivier Boulard, Mark J. Asquith, Fiona Powrie and Kevin J. Maloy

© 2010 WILEY-VCH Verlag GmbH & Co. KGaA, Weinheim

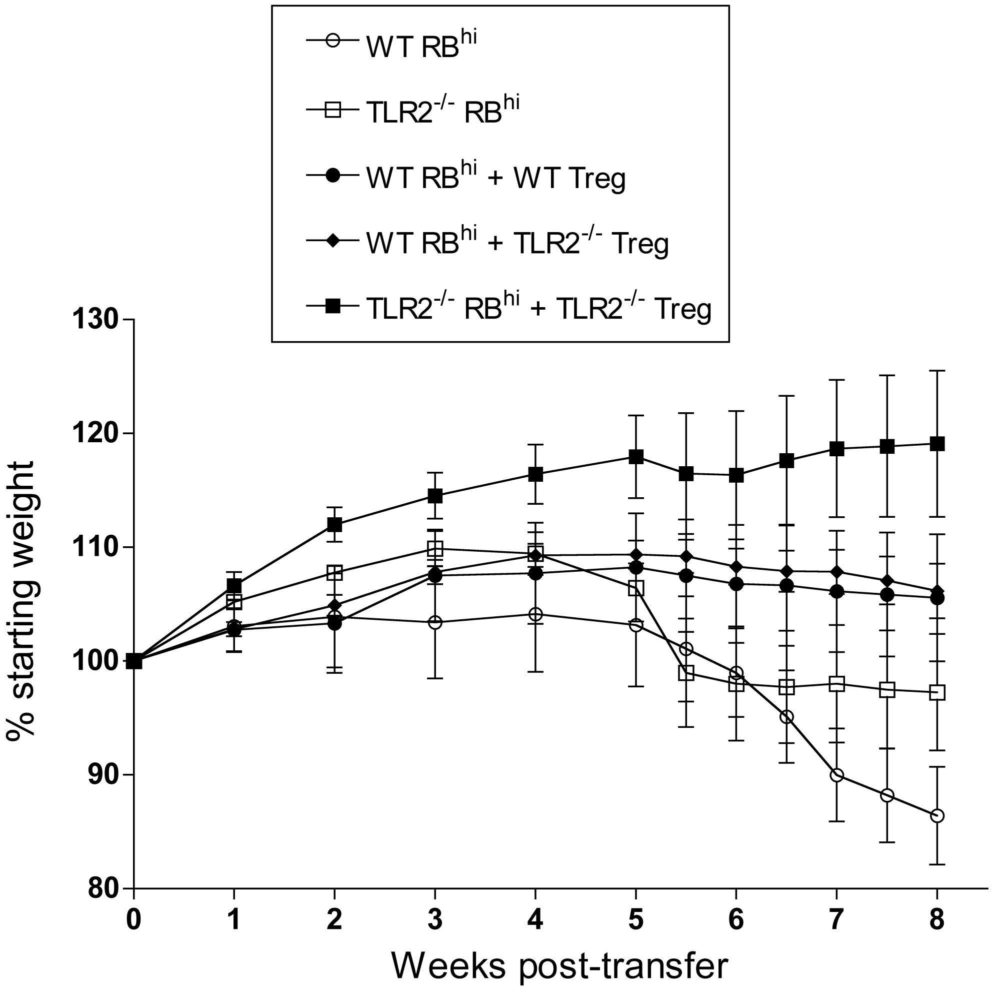

Supplementary Figure 1: TLR2 deficient effector and regulatory CD4$^+$ T cells mediate induction or prevention of wasting disease with similar kinetics as WT CD4$^+$ T cells. Cohorts of B6.RAG$^{-/-}$ mice were reconstituted with either 4 x 10^5 WT or TLR2$^{-/-}$ CD4$^+$CD45RB^{hi} T cells alone or concurrently with 2 x 10^5 WT or TLR2$^{-/-}$ CD4$^+$CD25$^+$ Treg cells. The onset of wasting disease was monitored by weighing the mice throughout the course of the experiment. Data shown represents group means (±SEM) of two pooled independent experiments (n=10-14 mice per group).
